# Friedewald’s equation for calculating LDL-cholesterol: Is it the time to say “Goodbye” and adopt direct LDL cholesterol methods?

**DOI:** 10.12669/pjms.35.2.679

**Published:** 2019

**Authors:** Sikandar Hayat Khan, Najmusaqib Khan Niazi, Farah Sobia, Nadeem Fazal, Syed Mohsin Manzoor, Ahmed Nadeem

**Affiliations:** 1 *Dr. Sikandar Hayat Khan, FCPS, PgD Endocrinology & Diabetes (UK). Department of Pathology, PNS Hafeez Hospital, Islamabad, Pakistan*; 2 *Dr. Najmusaqib Khan Niazi, MBBS, MSc. Healthcare Administration, PNS Hafeez Hospital, Islamabad, Pakistan*; 3 *Dr. Farah Sobia, FCPS. Department of Surgery, CMH Multan, Pakistan*; 4 *Nadeem Fazal, FCPS. Department of Medicine, PNS Hafeez Hospital, Islamabad, Pakistan*; 5 *Syed Mohsin Manzoor, FCPS. Department of Pathology, PNS Hafeez Hospital, Islamabad, Pakistan*; 6 *Ahmed Nadeem Medical Student, Agha Khan University, Karachi, Pakistan*

**Keywords:** Urine albumin creatinine ration (UACR), measured LDL-cholesterol (mLDLc), Calculated LDL-cholesterol (cLDLc), Friedewald’s equation

## Abstract

**Objectives:**

To measure correlation and concordance between measured LDL cholesterol (mLDLc) and Friedewald’s calculated LDL cholesterol (cLDLc). To compare the mLDLc and cLDLc values for various anthropometric measures and biochemical indices including insulin resistance, nephropathy, glycated hemoglobin and triglycerides.

**Methods:**

Two hundred thirty two subjects were included in this cross-sectional analysis from Jan-2016 to July-2017 from a target population visiting PNS HAFEEZ hospital. Mean age of the subjects was 46.56(±11.95) years (n=232). These subjects underwent clinical evaluation including measurement of anthropometric measurements, biochemical testing for fasting plasma glucose (FPG), glycated hemoglobin (HbA1c), lipid profile, urine albumin creatinine ratio (UACR), and insulin. Correlation and concordance between mLDLc and Friedewald’s cLDLc were measured. Finally, Comparison of risk evaluation for mLDLc and cLDLc between groups formulated based upon UACR (Based upon a cut off of 2.5 mg/g) and fasting triglycerides (Group-1 :< 1.0 mmol/L, Group-2: 1.0-1.99 mmol/L and Group-3 :> 1.99 mmol/) was carried out.

**Results:**

There was significant positive linear correlation between mLDLc and cLDLc [r=0.468, <0.001]. Kendall’s Coefficient of concordance between mLDLc and cLDLc was 0.055 (p<0.001). Differences evaluated by one way ANOVA analysis for mLDLc between various triglycerides groups were only significant between group-1 and group-2 [{Group-1:Mean=2.40, (2.19-2.61), n=43}, {Group-2:Mean=2.81, (2.69-2.92), n=136}, [{Group-3:Mean=2.59,(2.37-2.81), n=53}],(p=0.004) in comparison to cLDLc [{Group-1:Mean=2.63, (2.43-2.84), n=43}, {Group-2:Mean=2.85, (2.76-2.93), n=136}, [{Group-3:Mean=2.75, (2.60-2.90), n=53}]. Calculated method for LDLc showed higher UACR than mLDLc. (p=0.021)

**Conclusion:**

cLDLc over estimates LDL-cholesterol in comparison to mLDLc. The correlation between cLDLc and mLDLc was only moderate. However, cLDLc provided better degree of risk prediction for nephropathy and glycated hemoglobin than mLDLc.

## INTRODUCTION

The science of lipidology from the earliest work of Nikolai Antischkow have expanded its tentacles to various lipid and lipoprotein biomarkers for depicting risk for atherosclerotic cardiovascular diseases (ASCVD).^1^ Alongside the science also evolved to stratify and quantify the nature and potency of various lipid biomarkers including total cholesterol, triglycerides, LDL-cholesterol(LDLc) and HDL-cholesterol(HDLc).^2^ Two important lipid related risk assessment emerged for the assessment of ASCVD, which include the villainous one (LDL-cholesterol) and the good HDL-cholesterol.^3^

Earlier biotechnology did not provide clinically feasible solutions for lipoprotein measurements. Friedewald et al emerged on the clinical horizon as a panacea by simplifying LDLc measurements through a calculated method which involved total cholesterol, triglycerides and HDLc.^4^ Though criticism and limitations in terms of requirement of fasting sample, unsuitability for chylous specimens and Frederickson classified type-I hypercholesterolemia were the major limitations for this calculated LDLc,^5^ still the calculated LDLc (cLDLc) got slowly incorporated into the labs and received recommendations from various authorities.^6^,^7^ Alongside the directly measuring clinically suitable technologies for measuring LDLc also evolved, became simplified by removing multiple steps and to some extent cost-effective and less labor intensive. Current trend within labs depicts more shift towards directly measurement techniques of mLDLc (measured LDLc). Though mLDLc technologies seems more promising for highlighting underlying ASCVD, still erroneous results in presence of high triglycerides and related technical limitations downgrade the clinical yield of these slightly expensive methodologies.^8^-^10^ Moreover, many regional and new equations have emerged which support a more valuable use of indirect LDLc calculation methods.^11^-^14^ Therefore, in comparative terms adopting an accurate calculation based method for cLDLc can be less labor-intensive and cost-effective but will laboratory reporting more precise.

With this background information the study the correlation and concordance between calculated LDL (cLDLc), measured LDL (mLDLc). The objective was to study the difference of these measured and calculated LDL methods within groups formulated based upon glycemic status, Insulin Resistance status, nephropathy status and metabolic syndrome.

## METHODS

This cross-sectional study was conducted at PNS HAFEEZ hospital (Islamabad) in liaison with department of chemical pathology and clinical endocrinology, Armed Forces Institute of Pathology (AFIP) from January-2016 to July-2017. Our target population were adult subjects who were referred to the department of pathology in fasting for the evaluation of fasting plasma lipid profile. Subject selection was based upon “non-probability convenience sampling”. Subjects with chronic/acute disorder, pregnancy, indoor cases, using any medication were not included in the study. Subjects were formally consented after explanation of study related requirements and use of data for publication purpose. All finally selected participants signed a written consent form as per the hospital’s ethical review committee. After enrollment, subjects were interviewed as per a questionnaire and general clinical evaluation was carried out. Anthropometric indices including height, weight, waist and hip circumference were measured as per WHO protocol.^15^

Blood (10 ml approx.) was drawn from finally selected subjects (n=232) in plain bottles, EDTA and in Na-Fluoride tubes for measuring various biochemical parameters. Fasting plasma glucose (FPG) was measured by GOD-PAP method, glycated hemoglobin by fast ion-exchange resin separation method and serum insulin by chemiluminescence’s technique on Immulite® 1000. CHOD-PAP and GPO-PAP methods were used to measure cholesterol and triglycerides. mLDLc and HDLc were measured by cholesterol esterase method on ADVIA 1800 clinical chemistry system. Samples were urine albumin creatinine ratio (UACR) were collected for 174 subjects and analysis was carried out on imunoturbidimetric method on ADVIA 1800. Homeostasis Model Assessment for insulin resistance (HOMA-IR) was calculated as per the method of Mathew’s et al.^16^ LDL-cholesterol was measured using Friedewald’s formula.^4^ During specimen processing few samples were lost due to technical reasons including hemolysis, insufficient quantity and lack of patient follow up for repeat testing.

Grouping was done as:

1-Triglycerides related groups were as: Group-1(Fasting triglycerides: <1.0 mmol/L), Group-2 (Fasting triglycerides: 1.0-1.99 mmol/L) and Group-3 (Fasting triglycerides: >1.99 mmol/L)
2-UACR groups classified as: Group-1: < 2.5 mg/g and Group-2: >2.4 mg/g.

### Data Analysis

All data was entered into Excel software and later transferred to SPSS-15. Descriptive statistics for all parameters were calculated in terms of mean and standard deviation. Graphs were generated by employing both Excel and SPSS software.

Pearson’s correlation was utilized to correlation between mLDLc and cLDLc and also between other biochemical biomarkers. Kendall’s Coefficient of concordance method was utilized to see the concordance between mLDLc and cLDLc. One way ANOVA along with Tukey’s post-hoc analysis was used to compare mLDLc and cLDLc between various groups formulated based upon fasting triglycerides levels. Paired-t statistics was used to compare the mLDLc and cLDLc between nephropathy groups as assessed by UACR.

## RESULTS

**Fig.1:**
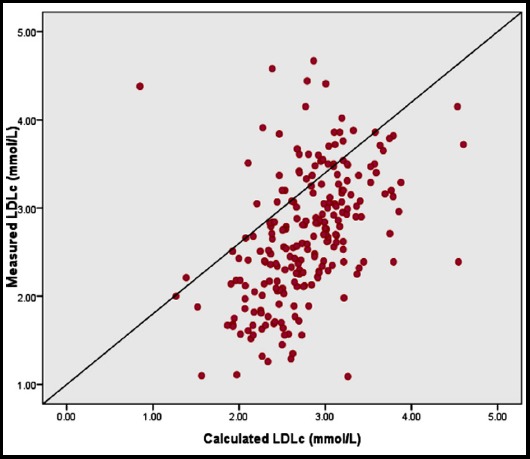
Correlation between measured LDL cholesterol (mLDLc) and calculated LDL cholesterol (cLDLc) [Pearson correlation coefficient (r) = 0.468, <0.001].

**Fig.2:**
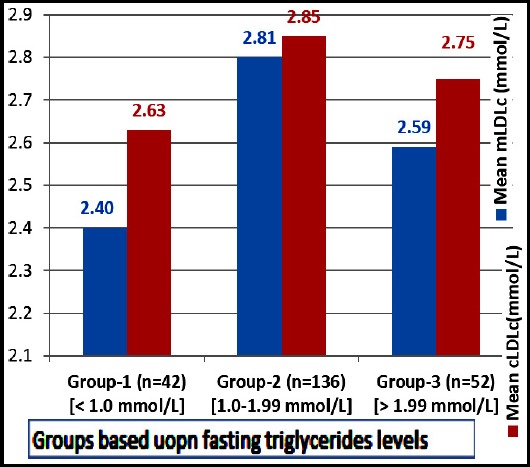
Histogram demonstrating differences in mean mLDLc and cLDLc among fasting triglycerides groups.

**Fig.3:**
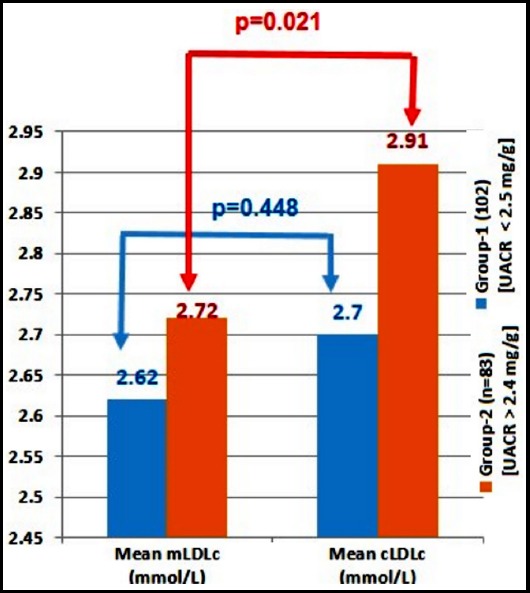
Histogram demonstrating differences in mean mLDLc and cLDLc among patients with UACR and low UACR (n=174).

Descriptive statistics are shown in Table-I. There were 122 females and 110 males. There was significant positive linear correlation between mLDLc and cLDLc as depicted in Fig.1. The correlation between biochemical parameters demonstrated and measured and calculated methods of LDL is shown in Table-II. The concordance between mLDLc and cLDLc as measured by Kendall’s Coefficient of concordance is shown in Table-III. One way ANOVA followed by Tukey’s post-hoc analysis between various groups formulated based upon fasting triglycerides levels only demonstrated significance differences (p=0.004) between group-1 and group-2 for mainly mLDLc [{Group-1:Mean=2.40, (2.19-2.61), n=43}, {Group-2:Mean=2.81, (2.69-2.92), n=136}, [{Group-3:Mean=2.59, (2.37-2.81), n=53}] in comparison to cLDLc [{Group-1:Mean=2.63, (2.43-2.84), n=43}, {Group-2:Mean=2.85, (2.76-2.93), n=136}, [{Group-3:Mean=2.75, (2.60-2.90), n=53}]. (Fig.2) Fig.3 demonstrates calculated method for LDLc to be higher among subjects with nephropathy in comparison to mLDLc.

**Table-I:** Descriptive statistics for data.

Parameters	n	Mean		Std. Deviation

		Statistic	Std. Error	
Age (years)	232	46.56	0.785	11.95
Body Mass Index(BMI)	232	27.13	0.35	5.22
Waist to hip ratio (WHpR)	232	0.93	0.043	0.08
Fasting plasma Glucose (mmol/L)	232	5.62	0.148	2.26
Total cholesterol (mmol/L)	232	4.48	0.039	0.61
Triglycerides (mmol/L)	232	1.61	0.049	0.75
HDLc (mmol/L)	230	0.98	0.017	0.26
mLDLc (mmol/L)	230	2.68	0.048	0.72
cLDLc (mmol/L)	232	2.79	0.036	0.55
HbA1c (%	228	5.76	0.064	0.97
Homeostasis Model Assessment for Insulin Resistance (HOMA-IR)	228	2.83	0.256	3.86
UACR (mg/g)	174	2.74	0.370	4.90

**Table-II:** Pearson correlation between various biochemical risk biomarkers and mLDLc and cLDLc.

Parameter	Statistics	cLDLc (mmol/L)	mLDLc (mmol/L)
HOMA-IR	Pearson Correlation	-0.032	-0.035
	Sig. (2-tailed)	0.627	0.598
	n	228	227
UACR (g/mg)	Pearson Correlation	0.083	0.098
	Sig. (2-tailed)	0.276	0.197
	n	174	173
HbA1c (%)	Pearson Correlation	-0.132^*^	-0.011
	Sig. (2-tailed)	0.046	0.864
	n	228	226
Insulin (mIU/L)	Pearson Correlation	0.026	0.001
	Sig. (2-tailed)	0.696	0.989
	n	228	227

**Table-III:** Concordance between mLDLc and cLDLc.

Parameter	n	Mean	Std. Deviation	Kendall’s Coefficient of concordance	Asymp. Sig. (p-Value)
mLDLc (mmol/L)	230	2.68	0.723	0.055	<0.001
cLDLc (mmol/L)	230	2.78	0.538		

## DISCUSSION

Our study has shown significant positive correlation and concordance between mLDLc and cLDLc. However, the results between the two LDLc measures remain significantly different with regards to association with evaluated biochemical parameters implying that two parameters act to measure different aspects of disease. There is much evidence supporting the observed differences between mLDLc and cLDLc in literature. An earlier Pakistani study by Fawwad A et al showed triglyceride related bias, with recommendations for using direct methods.^17^ Jun KR et al in a recent study has demonstrated that Fiedewld’s equation may underestimate coronary heart disease in comparison to direct methods.^18^ Another study from Iran showed strong correlation between the calculated and measured LDLc but highlighted that Friedewald’s equation overestimate LDLc.^19^ In contrasts to above finding literature Kamezaki F et al have concluded that direct measuring LCLc methods over estimates, rather than underestimates LDLc results.^20^ Chotkowska et al have also highlighted mLDLc methods once compared with gold standard technique of ultracentrifugation to demonstrate a positive bias in comparison to Friedewald’s equation.^21^

Taken together the contrasts in literature with regards to methodologies over and underestimating triglycerides, possible explanation could be: First of all, as the triglyceridemia affects LDLc measures in calculated methods which is evident by the use of different factors to manage VLDL in serum triglyceride calculation, so calculated methods are inherently biased to the presence of triglyceride adjustment.^22^,^23^ Secondly, variation between LDL associations with other biochemical risks and between mLDLc and cLDLc may be attributed to differences measuring technologies as Kamezaki F et al and Chotkowska et al have utilized ultra-centrifugation methods in contrasts our methodology.^17^-^21^ Finally, regional differences have been highlighted by multiple studies, and thus it becomes pertinent to interpret our LDLc calculations with the perspective of interpreting racial and regional factors. However, we feel as authors that the differences based upon race and regions are marginal and will not final affect final interpretation of the data.^24^,^25^

### Limitations of the study

This was a cross-sectional study which was meant to highlight only association between mLDLc and cLDLc, where we understand that various other homogenous methods are now available to measure LDLc directly. So results are bound to be different between different methodologies. Secondly, the sample size is small which can lead to possible type-2 statistical error. A randomized controlled study with more sample size incorporating various available homogenous mLDLc should be carried out to augment or refute our findings.

The study is considered clinically important as decades old Friedewald’s equation for LDLc calculation looked destined to end up as an obsolete lipid biomarker, but the current study provides some life to cLDLc on account of moderate correlation, concordance, and slight degree of association in terms of nephropathy and with diabetogenic tendencies. Being cost-effective, least labor intensive and with aforementioned statistical information from our study we feel it will still remain useful in developing economies for some time to come.

## CONCLUSION

Though cLDLc have shown concordance with mLDLc along with moderate correlation and has been proven to depict some degree of lipid related risk for nephropathy and diabetic indices, still cLDLc over estimates LDLc in comparison to mLDLc. However, till the homogenous methods are not fully evolved in biotechnology and become cost-effective, cLDLc can be useful in lipid associated risk prediction.

### Authors’ Contribution

**SHK, SMM:** Conceived, designed, SPSS analysis, discussion and results.

**NKZ, FS, NF, AN:** Data collection, sampling and manuscript writing.

**SHK:** Final review as well.

## References

[1] Anitschkow N (1913). Ueber die Veranderungen der Kaninchenaorta bei experimenteller Cholesterinsteatose. Beitr Pathol Anat.

[2] Siri-Tarino PW, Krauss RM (2016). The early years of lipoprotein research:from discovery to clinical application. J Lipid Res.

[3] Gofman JW, Glazier F, Tamplin A, Strisower B, De Lalla O (1954). Lipoproteins, coronary heart disease, and atherosclerosis. Physiol Rev.

[4] Friedewald WT, Levy RI, Fredrickson DS (1972). Estimation of the concentration of low-density lipoprotein cholesterol in plasma, without use of the preparative ultracentrifuge. Clin Chem.

[5] Campos EM (2005). From Fredrickson's classification of phenotypes--lipoprotein patterns--to genotype comprehension. Acta Med Port.

[6] Hoerger TJ, Wittenborn JS, Young W (2011). A cost-benefit analysis of lipid standardization in the United States. Prev Chronic Dis.

[7] Whelton SP, Meeusen JW, Donato LJ, Jaffe AS, Saenger A, Sokoll LJ (2017). Evaluating the atherogenic burden of individuals with a Friedewald-estimated low-density lipoprotein cholesterol <70 mg/dL compared with a novel low-density lipoprotein estimation method. J Clin Lipidol.

[8] Schaefer EJ, Otokozawa S, Ai M (2011). Limitations of direct methods and the reference method for measuring HDL and LDL cholesterol. Clin Chem.

[9] Miller WG, Myers GL, Sakurabayashi I, Bachmann LM, Caudill SP, Dziekonski A (2010). Seven direct methods for measuring HDL and LDL cholesterol compared with ultracentrifugation reference measurement procedures. Clin Chem.

[10] Kim SJ, Park YG, Kim JH, Han YK, Cho HK, Bang OY (2012). Plasma fasting and nonfasting triglycerides and high-density lipoprotein cholesterol in atherosclerotic stroke:different profiles according to low-density lipoprotein cholesterol. Atherosclerosis.

[11] Kapoor R, Chakraborty M, Singh N (2015). A Leap above Friedewald Formula for Calculation of Low-Density Lipoprotein-Cholesterol. J Lab Physicians.

[12] Martin SS, Blaha MJ, Elshazly MB, Toth PP, Kwiterovich PO, Blumenthal RS (2013). Comparison of a novel method vs the Friedewald equation for estimating low-density lipoprotein cholesterol levels from the standard lipid profile. JAMA.

[13] de Cordova CM, de Cordova MM (2013). A new accurate, simple formula for LDL-cholesterol estimation based on directly measured blood lipids from a large cohort. Ann Clin Biochem.

[14] Puavilai W, Laorugpongse D, Deerochanawong C, Muthapongthavorn N, Srilert P (2009). The accuracy in using modified Friedewald equation to calculate LDL from non-fast triglyceride:a pilot study. J Med Assoc Thai.

[15] (2008). Waist circumference and waist-hip ratio:Report of a WHO expert consultation.

[16] Matthews DR, Hosker JP, Rudenski AS, Naylor BA, Treacher DF, Turner RC (1985). Homeostasis model assessment:insulin resistance and beta-cell function from fasting plasma glucose and insulin concentrations in man. Diabetologia.

[17] Fawwad A, Sabir R, Riaz M, Moin H, Basit A (2016). Measured versus calculated LDL-cholesterol in subjects with type 2 diabetes. Pak J Med Sci.

[18] Jun KR, Park HI, Chun S, Park H, Min WK (2008). Effects of total cholesterol and triglyceride on the percentage difference between the low-density lipoprotein cholesterol concentration measured directly and calculated using the Friedewald formula. Clin Chem Lab Med.

[19] Boshtam M, Ramezani MA, Naderi G, Sarrafzadegan N (2012). Is Friedewald formula a good estimation for low density lipoprotein level in Iranian population?. J Res Med Sci.

[20] Kamezaki F, Sonoda S, Nakata S, Otsuji Y (2010). A direct measurement for LDL-cholesterol increases hypercholesterolemia prevalence:comparison with Friedewald calculation. J UOEH.

[21] Chotkowska E, Kurjata P, Kupsc W (2001). Evaluation of the precision of the Friedewald's formula for the calculation of low density lipoprotein cholesterol concentration in serum. Pol Merkur Lekarski.

[22] Chowdhury N, Saiedullah M, Khan MA, Rahman MR (2013). Comparison of modified Friedewald's formula with direct measurement of low-density lipoprotein cholesterol in Bangladeshi population. Bangladesh Med Res Coun Bull.

[23] Dansethakul P, Thapanathamchai L, Saichanma S, Worachartcheewan A, Pidetcha P (2015). Determining a new formula for calculating low-density lipoprotein cholesterol:data mining approach. EXCLI J.

[24] Patel SA, Shivashankar R, Ali MK, Anjana RM, Deepa M, Kapoor D (2016). CARRS Investigators. Is the “South Asian Phenotype”Unique to South Asians?Comparing Cardiometabolic Risk Factors in the CARRS and NHANES Studies. Glob Heart.

[25] Santos RD, Bensenor IM, Pereira AC, Lotufo PA (2016). Dyslipidemia according to gender and race:The Brazilian Longitudinal Study of Adult Health (ELSA-Brasil). J Clin Lipidol.

